# Mycological Survey of Fungal Carriage in Cats and Their Owners: Analysis of Species Diversity and Antifungal Susceptibility

**DOI:** 10.3390/jof12040245

**Published:** 2026-03-26

**Authors:** Kittima Siripit, Naris Thengchaisri, Orawan Limsivilai, Sara Niae, Panpicha Sattasathuchana, Chompoonek Yurayart

**Affiliations:** 1 Department of Microbiology and Immunology, Faculty of Veterinary Medicine, Kasetsart University, Bangkok 10900, Thailand; kittima.sir@ku.th (K.S.); orawlim826@gmail.com (O.L.); 2 Department of Companion Animal Clinical Sciences, Faculty of Veterinary Medicine, Kasetsart University, Bangkok 10900, Thailand; ajnaris@yahoo.com (N.T.); psatta99@gmail.com (P.S.); 3 Faculty of Veterinary Science, Rajamangala University of Technology Srivijaya, Nakhon Si Thammarat 80240, Thailand; sara.n@rmutsv.ac.th

**Keywords:** cat, human, dermatophytosis, carriage, zoonotic

## Abstract

Healthy and asymptomatic cats may serve as reservoirs of fungal pathogens, facilitating transmission through direct contact or environmental contamination, and they may represent an underrecognized source of subclinical fungal infection in humans, particularly among cat owners and veterinarians. We evaluated the prevalence of fungal species in healthy cats and their owners, assessed potential cat–human transmission, identified feline lifestyle factors associated with *Microsporum canis* carriage, and evaluated antifungal susceptibility of the most prevalent isolated fungi. We collected 59 cat facial hair and 59 owner nail samples for fungal isolation and identification. Five fungal species were identified, *M. canis*, *Aspergillus flavus*, *A. niger*, *A. fumigatus*, and *A. terreus*, which were found in both cats and humans. *Aspergillus* spp. were the most frequently detected fungi in both groups. Significant associations between cats and owners were observed for *M. canis* (*p* = 0.010) and *A. niger* (*p* = 0.050). Long-haired cats showed a significantly higher prevalence of carrying *M. canis* (*p* = 0.024), while other lifestyle factors were not associated with fungal carriage. The antifungal susceptibility profiles of the tested fungi were broadly similar between feline and human isolates; however, resistance to itraconazole and amphotericin B was detected among *Aspergillus* spp. Healthy cats and their owners frequently share fungal species, especially *M. canis*, which suggests possible household zoonotic transmission. Long-haired cats are at higher risk of *M. canis* carriage.

## 1. Introduction

Dermatophytosis is a superficial fungal infection caused by keratinophilic fungi. It is a zoonotic disease transmitted from animals to humans. It significantly impacts both animal health and public hygiene [1]. The main fungal genera that cause dermatophytosis are *Trichophyton*, *Epidermophyton*, and *Microsporum* [2,3], with cats being the most important reservoir for *M. canis* [4,5]. These fungi can be detected in healthy or asymptomatic cats, which act as carriers and spread the infection through direct contact with humans and other animals or via environmental contamination, increasing the risk of infection for pet owners and veterinary personnel [6,7]. Cats are popular pets worldwide and provide mental health benefits to their owners, including improved mental health and increased happiness [8]. However, cats can pose public health risks through zoonotic diseases, such as dermatophytosis [9,10,11]. Fungal spread is influenced by geographical and climatic conditions, with warm and humid tropical regions favoring growth [12]. Additionally, cat-related factors, including age, coat length, and lifestyle, may influence the incidence of this disease [7].

The treatment of dermatophytosis typically involves a combination of topical and systemic antifungal therapies. However, the delicate nature of feline skin can limit the use of certain topical agents due to irritation or side effects. The inconsistent application of treatments by owners can also contribute to disease recurrence [4], and the widespread use of antifungal products raises concerns about emerging antifungal resistance [13]. Previous studies show that yeast can be shared between cats and humans in the same household, with analyses of cat facial hair and human nails indicating potential cross-contamination among pets, people, and the environment [14]. Although several studies have reported dermatophyte carriage in cats, particularly *M. canis* [6,7,11], data on dermatophyte carriage in healthy indoor cats, their role in household transmission, and the relationship between fungal species isolated from cats and their owners remain limited. We hypothesize that healthy, asymptomatic cats act as subclinical carriers of dermatophytes, particularly *M. canis*, and may contribute to human exposure within the same household. In addition, the influence of lifestyle factors and antifungal susceptibility patterns also remains poorly understood. Data on antifungal susceptibility in dermatophytes obtained from healthy cats and their owners are limited, suggesting the need for further study regarding the transmission dynamics within shared human–cat environments.

This study was intended to investigate the prevalence and distribution of fungal species in healthy cats and their owners living in the same household, assess potential cat–human transmission, evaluate the feline lifestyle factors associated with *M. canis* carriage, and determine the antifungal susceptibility profiles of fungal isolates to better inform the prevention and control of zoonotic fungal infections.

## 2. Materials and Methods

### 2.1. Ethical Considerations

This study continues the comparative analysis of the distribution and antifungal susceptibility of yeast species in cat facial hair and human nails by investigating the involvement of fungi in healthy pet cats and humans [14]. The study involving human participants was approved by the Ethics Committee of Kasetsart University (Approval No. COE#66/014, approved on 16 February 2023). Animal procedures were approved by the Kasetsart University Institutional Animal Care and Use Committee (IACUC) (Approval No. ACKU66-VET-041, approved on 30 May 2023). Written informed consent was obtained from all participants, and cat owners provided informed consent on behalf of their pets. All procedures complied with relevant ethical guidelines and regulations.

### 2.2. Sample Collection

**Figure 1:**
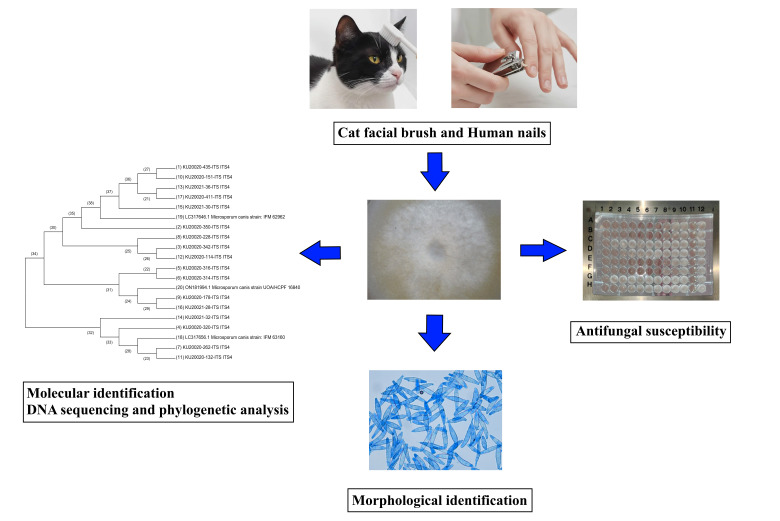
Schematic representation of the study design. Samples collected from cat subjects and their owners underwent fungal culture and molecular identification using fungal DNA barcoding based on amplification and sequencing of the internal transcribed spacer (ITS) region of ribosomal DNA.

All samples were collected at Kasetsart University Veterinary Teaching Hospital, in Bangkok, Thailand. A total of 118 biological samples were collected, including 59 facial hair samples from cats and 59 nail samples from their owners (Figure 1). Based on owner-reported information from the questionnaire, cats were classified as either indoor-only or having outdoor access. Indoor cats were defined as cats kept exclusively inside the household without access to outdoor environments. Cats with outdoor access were those allowed to go outside the house or into the surrounding environment. Most cats were kept exclusively indoors (*n* = 52), whereas seven cats had outdoor access. The cat owners completed a questionnaire and provided fingernail samples by trimming their own nails. These nail samples were collected from the cat owners only. All participating cat owners were residents of an urban area in Bangkok, Thailand, and none were engaged in farming activities. For each cat, data on age, sex, breed, body weight, body condition score, and hair length were recorded. Hair length and age were each categorized into two groups (≤3 cm and >3 cm for hair length; ≤6 years and >6 years for age) to distinguish short- and long-haired cats and to allow comparison between younger and older animals while maintaining sufficient sample size for statistical analysis. Each cat underwent a physical examination and a complete blood count, which was performed using a Sysmex XN-1000TM hematology analyzer (Sysmex, IL, USA), to assess health status. During the examination, none of the cats showed visible dermatological lesions. The cats were not followed after sampling; therefore, subsequent development of dermatological lesions could not be evaluated. A veterinarian collected facial hair and skin scales from each cat using a sterile brush. The brush was applied sequentially to the following areas: from the end of the nostril to the forehead, from the forehead to the occipital region, from the lip to the mandibular joint, and from the lip to the chin. All cats were seronegative for feline leukemia virus antigen and feline immunodeficiency virus antibodies, as determined by a commercial rapid immunochromatographic test (Witness^®^, Lyon Cedex, France).

### 2.3. Identification of Fungal Isolates

Fungal culture and phenotype identification: cat facial hair and human nail samples were inoculated on potato dextrose agar (PDA; Difco, Detroit, MI, USA) containing oxytetracycline (50 mg/L; Oxycline, Bangkok, Thailand) to facilitate mold culture and isolation. The agar plates were incubated at 30 °C and examined every 3 days over a period of 21 days. Fungal species are identified using morphological characteristics, including an examination of colony morphology at both the macroscopic and microscopic levels. The key microscopic structures assessed are septate hyphae, sporulation patterns, and conidiophore characteristics. These observations are then compared with the academic literature to identify the fungi at the genus or species level. The most prevalent fungal isolates were stored at 25 °C in distilled water stocks for future antifungal susceptibility testing. Additionally, fungal cells were harvested and stored at −20 °C for species confirmation using molecular identification.

### 2.4. Molecular Identification

Representative isolates from each genus and species, which were selected based on their distinct morphologies, were subjected to species confirmation using fungal DNA barcoding [15]. The genomic DNA was extracted from the isolates using a previously described method [16]. In brief, fungal samples were frozen at −20 °C overnight and then ground with a mortar and pestle in liquid nitrogen. The ground cells were then mixed with lysis buffer (0.5 g of sodium dodecyl sulfate; Himedia, Mumbai, India), NaCl (1.4 g; Carlo Erba, MI, USA), disodium ethylenediaminetetraacetic acid (0.73 g; Biobasic, Ontario, Canada), 1 M Tris–HCl (20 mL; Biobasic, Ontario, Canada), and 2-mercaptoethanol (5 µL; Sigma Aldrich, Steinheim, Germany) and incubated at 65 °C for 1 h with occasional mixing. Lysates were extracted with phenol-chloroform-isoamyl alcohol (25:24:1, *v*/*v*/*v*), and the DNA was precipitated using cold isopropanol. The internal transcribed spacer (ITS) region of ribosomal DNA was amplified using the SR6R (5′-AAGTATAAGTCGTAACAA GG-3′) and ITS4 (5′-TCCTCCGCTTATTGATATGC-3′) primer sets. Amplicons were verified by electrophoresis on a 1.2% agarose gel. All PCR products were purified and sequenced in both the forward and reverse directions using ABI BigDye Terminator V.3.1 (Macrogen Inc., Seoul, Korea). Sequences were assembled and manually edited for consistency using BioEdit v7.2.5 software (https://bioedit.software.informal.com). ITS sequence similarity and species identification were determined using BLASTn, which was obtained from the National Center for Biotechnology Information (http://blast.ncbi.nlm.nih.gov/Blast.cgi, accessed on 20 March 2026). The gene sequences of all representative isolates were submitted to GenBank (accession numbers are provided in Supplementary Table S1).

### 2.5. In Vitro Antifungal Susceptibility Testing

In vitro antifungal susceptibility was evaluated against *M. canis* and *Aspergillus* spp., the most prevalent fungal species affecting humans and cats, using the broth microdilution method according to Clinical and Laboratory Standards Institute (CLSI) guideline M38-A for filamentous fungi [17]. Antifungal agents, including amphotericin B (AMB), itraconazole (ITZ), ketoconazole (KZ), and terbinafine (TERB), all of which were sourced from Sigma-Aldrich (MO, USA), were dissolved in dimethyl sulfoxide and diluted in RPMI 1640 culture medium (Invitrogen, Waltham, MA, USA) buffered to a pH of 7.0 with 0.65 M MOPS buffer (Sigma, St. Louis, MO, USA) with L-glutamine and without bicarbonate prior to addition to 96-well round-bottom microdilution plates (Corning Costar, Corning Incorporated, Kennebunk, ME, USA). Final concentrations ranged from 0.06 to 32 µg/mL for AMB and ITZ and from 0.015 to 8 µg/mL for KZ and TERB. Antifungal susceptibility testing for *M. canis* was performed according to CLSI M38-A, with modifications [18]. Briefly, inocula were obtained from 7-day-old colonies grown on PDA at 30 °C. Hyphae and conidia were gently scraped from the colony surface, mixed in sterile saline solution, and filtered through sterile filter paper (Whatman filter, model Grade 40, pore size 8 µm). Cell density was measured via spectrophotometry at 530 nm, adjusted to 70–72% transmittance, and diluted in RPMI (1:50) to achieve a final cell concentration of 0.4 to 5 × 10^4^ CFU/mL. For *Aspergillus* spp., the broth microdilution test was performed according to CLSI M38-A. In brief, inocula were obtained from a 7-day-old culture grown on PDA at 30 °C. Conidia were collected using a sterile swab and stirred in sterile normal saline before being allowed to settle for 10–15 min at room temperature. The upper part of the suspension was measured with a spectrophotometer at 530 nm, adjusted to 0.09–0.11, and diluted, as described in the *M. canis* cell preparation procedure [19]. The minimum inhibitory concentration (MIC) was determined by naked-eye observation after 3 days of incubation at 35 °C for *M. canis* and 48 h for *Aspergillus* spp. The MICs of AMB and ITZ were defined as the lowest concentrations that inhibited 100% growth relative to an inoculum-free medium control. For TERB and KZ, the MICs were defined as the lowest concentration that inhibited growth by 80% relative to a drug-free growth control. *Candida parapsilosis* ATCC 22019 was included as a quality control strain.

### 2.6. Statistical and Data Analysis

The prevalence of each fungal organism on human nails and cat facial hair was calculated, as were corresponding 95% confidence intervals (CIs). The Shapiro–Wilk test was used to assess the normality of all data. Fisher’s exact test evaluated the association between fungal organisms in cats and humans residing in the same household (STATA12, StataCorp, College Station, TX, USA). The minimum inhibitory concentrations (MIC_50_ and MIC_90_) of the antifungal agents (AMB, ITZ, KZ, and TERB) were described and compared between cat and human fungal isolates. A *p*-value ≤0.05 was considered statistically significant. The non-wild-type (non-WT) isolates were identified as those *Aspergillus* spp. isolates with MIC values higher than the epidemiological cutoff values (ECVs) previously reported for AMB and ITZ [20,21].

## 3. Results

In total, 118 biological samples were obtained, including 59 facial hair samples from cats and 59 nail samples from their owners. A total of 59 cats were included in the study, with a mean age of 5.7 ± 3.7 years and an average body weight of 5.1 ± 1.5 kg. Thirty-seven were male and 22 were female. The most common breed was the domestic shorthair (DSH) (*n* = 27), followed by the Persian (*n* = 19), Maine coon (*n* = 4), mixed long-haired (*n* = 3), Bengal (*n* = 2), Scottish fold (*n* = 2), American wirehair (*n* = 1), and munchkin (*n* = 1). Most cats were kept exclusively indoors (*n* = 52), and only a few had outdoor access (*n* = 7). The participating cat owners had a mean age of 39.2 ± 11.5 years and included 45 males and 14 females. All participating cat owners were residents of an urban area of Bangkok, Thailand, and none reported engaging in farming activities.

**Figure 2:**
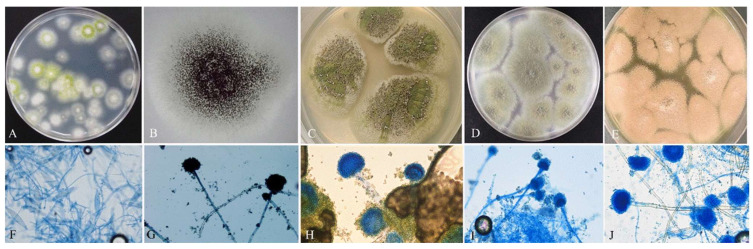
Macroscopic and microscopic identification of mold species, including *Microsporum canis* (**A**,**F**), *Aspergillus niger* (**B**,**G**), *Aspergillus flavus* (**C**,**H**), *Aspergillus fumigatus* (**D**,**I**), and *Aspergillus terreus* (**E**,**J**). The top row (**A**–**E**) shows fungal growth on culture plates, while the bottom row (**F**–**J**) provides a microscopic characterization of their reproductive structures stained with lactophenol cotton blue.

Based on morphological characteristics, *M. canis* and four species of *Aspergillus* spp. were identified; as illustrated in Figure 2, the macroscopic characteristics of *M. canis* colonies cultured on potato dextrose agar at 30 °C developed within 7 to 21 days, with initial growth being observed between days 3 and 5. Colonies initially appeared white, soft, and fluffy at the center, surrounded by yellow or golden-yellow margins and closely spaced radial grooves. Over a period of 3 to 4 weeks, the colonies became uniformly white. The reverse side displayed yellow pigmentation that gradually faded to brown and darkened with age. Microscopic analysis demonstrated that *M. canis* produced numerous spindle-shaped macroconidia with rough surfaces and knob-like ends and up to 11 septa when stained with lactophenol cotton blue. Microconidia were present in small numbers along the hyphae. They displayed a pear-shaped to round morphology. Among the 59 cats examined, *M. canis* was isolated from 17 animals (28.81%). Among the 59 cat owners, *M. canis* was isolated from four individuals (6.78%), yielding a total of 21 isolates.

In this study, two groups of fungi were identified: the dermatophytes group (*M. canis*) and the non-dermatophytes group (hyaline septate fungi, dematiaceous fungi, *Aspergillus* spp., *Penicillium* spp., and non-septate fungi). Four species—*M. canis*, *A. flavus*, *A. niger*, and *A. fumigatus*—were isolated from both cats and their owners (Table 1). In cats, the most frequently identified species was *Aspergillus* spp. (*n* = 39; 66.10%), followed by hyaline septate fungi (*n* = 32; 54.24%), dematiaceous fungi (*n* = 26; 44.07%), *M. canis* (*n* = 17; 28.81%), and *Penicillium* spp. (*n* = 10; 16.95%). The *Aspergillus* spp. included *A. flavus* (*n* = 23; 38.98%), *A. niger* (*n* = 26; 44.07%), *A. fumigatus* (*n* = 1; 1.69%), and *A. terreus* (*n* = 1; 1.69%). From human nail samples, five fungal species were similarly identified. *Aspergillus* spp. was the most common species (*n* = 27; 45.76%), followed by hyaline septate fungi (*n* = 21; 35.59%), dematiaceous fungi (*n* = 5; 8.47%), *M. canis* (*n* = 4; 6.78%), and *Penicillium* spp. (*n* = 2; 3.39%). The identified *Aspergillus* spp. included *A. flavus* (*n* = 15; 25.42%), *A. niger* (*n* = 17; 28.81%), and *A. fumigatus* (*n* = 1; 1.69%). Interestingly, the association analysis demonstrated significant correlations for *M. canis* (*p* = 0.010) isolated from the cat’s facial hair and the same fungus in the human’s nails, and between *A. niger* (*p* = 0.050) in both sources. No significant associations occurred for *A. flavus*, *A. fumigatus*, or *A. terreus* isolated from the same sample pairs (Table 2).

Because *M. canis* poses a zoonotic threat, further analysis was conducted to evaluate its association with feline lifestyle factors (Table 3). The results showed that long-haired cats (>3 cm) had a significantly higher prevalence of *M. canis* positivity (76.47%) as compared with short-haired cats (23.53%; *p* = 0.024). Among a group of long-haired cats, *M. canis* was predominantly detected in the Persian breed (*n* = 9/17), followed by mixed long-haired cats (*n* = 2/17) and Scottish fold cats (*n* = 1/17), while for short-haired cats, *M. canis* was solely detected in DSH cats (*n* = 5/17). Although cats bathed less frequently (>1 month or never) showed a higher positivity rate (58.82%) than those bathed monthly (41.18%), this difference was not statistically significant (*p* = 0.103). No significant associations were found with sex, age group, or rearing environment (indoor-only vs. outdoor access) (Table 3).

The influence of yeast on the presence of filamentous fungi was evaluated in both cats (Table 4) and humans (Table 5). In cats, the presence of *Malassezia pachydermatis* reported in our previous study [14] was not significantly associated with the isolation of any filamentous fungal species from facial hair or external ear canal samples, with no differences being observed for *M. canis*, *Aspergillus* spp., *Penicillium* spp., hyaline septate fungi, dematiaceous fungi, or non-septate fungi (*p* > 0.05, Table 4). Similarly, in humans, *Malassezia furfur* detected in our previous study [14] was not associated with most filamentous fungi in nail samples, including *M. canis*, *Penicillium* spp., hyaline septate fungi, and dematiaceous fungi (*p* > 0.05); however, a significantly higher prevalence on the part of *Aspergillus* spp. was detected in *M. furfur*–positive samples as compared with negative samples (*p* = 0.0327, Table 5).

In vitro antifungal susceptibility testing for AMB, ITZ, KZ, and TERB was performed on fungal isolates derived from cats and humans living in the same households (Table 6). *M. canis* isolates from cats (*n* = 17) and humans (*n* = 4) showed comparable MIC_50_ and MIC_90_ values across all drugs. Similar MIC ranges and MIC_50_ and MIC_90_ values were observed for AMB, KZ, and TERB. However, the MIC_90_ for cat isolates was higher for ITZ (1 µg/mL) than for human isolates (ITZ 0.125 µg/mL). All identified *Aspergillus* spp., including *A. flavus*, *A. niger*, *A. fumigatus*, and *A. terreus*, exhibited similar antifungal susceptibility profiles for all testing drugs between cat- and human-originated isolates. All isolates of *A. flavus* and *A. niger* were defined as WT for AMB and ITZ; however, *A. flavus* exhibited a high MIC_90_ for AMB (2–4 µg/mL) as compared with ITZ (1 µg/mL), TERB (1 µg/mL), and KZ (1–2 µg/mL), while *A. niger* was more sensitive to AMB, with MIC_90_ at 1 µg/mL, but less susceptible to ITZ (MIC_90_ = 8 µg/mL), TERB (MIC_90_ = 4–8 µg/mL), and KZ (MIC_90_ = 4–8 µg/mL). *A. fumigatus* isolates obtained from cats and humans were similar in their response to AMB with WT phenotype but different for ITZ with WT and non-WT phenotypes for cat and human isolates, respectively. *A. fumigatus* showed high MICs for TERB (MIC = >8 µg/mL) and KZ (MIC = 4 µg/mL). One isolate of *A. terreus* recovered from a cat was defined as WT for ITZ (MIC = 1 µg/mL) but resistant to AMB and TERB (MIC = 16 µg/mL and 8 µg/mL, respectively).

## 4. Discussion

The present findings support the hypothesis that asymptomatic cats act as carriers of dermatophytes, spreading infection through direct contact or environmental contamination. In this study, the detection of dermatophytes in clinically healthy cats emphasizes their epidemiological importance as subclinical reservoirs rather than merely incidental hosts. The isolation of zoophilic dermatophytes such as *M. canis* from both cats and their owners indicates shared exposure within the household environment and supports the possibility of household-level transmission. These findings emphasize the zoonotic relevance of these fungi even in healthy or asymptomatic animals [6,7].

The isolation of multiple fungal species, especially *M. canis* and *Aspergillus* spp., from both cats and owners suggests shared exposure within the household, supporting the possibility of household-level transmission. Consistent with this observation, a previous study of 56 patients with cat-to-human dermatophytosis reported *M. canis* as the primary pathogen (83.9%), followed by *Trichophyton mentagrophytes* (16.1%) [22]. The observed prevalence of *M. canis* in subclinical cats in this study was higher than previously reported in Thailand (10.5–18.9%) [6,7], but similar to rates in other regions (22–35%) [23,24]. The sample collection method and anatomical site may influence fungal detection rates, and organism detection from one sampling location may not necessarily reflect those present at another site. Previous studies have shown that collecting samples from the head, ears, limbs, and whole body yielded higher rates than sampling from the trunk, back, or ventral abdomen [25,26]. In the present study, facial hair samples were collected from clinically healthy cats. This may explain the high detection rate of *M. canis*, as the facial region is frequently associated with dermatophyte colonization and grooming behavior [4], and may be appropriate for surveillance sampling in asymptomatic cats.

Cat lifestyle factors may influence fungal carriage. In this study, long-haired cats had a significantly higher prevalence of *M. canis* than short-haired cats. This supports the hypothesis that long hair coats facilitate the adherence and persistence of fungal spores along the hair shaft, thereby enhancing their transmission potential. This agrees with previous research on 127 privately owned indoor cats without dermatophytosis lesions. *M. canis* was significantly detected in long-haired cats, with Persian cats being the predominant breed affected [7]. Interestingly, no significant associations were observed with other factors, such as age, sex, bathing frequency, or rearing environment. These findings were consistent with those previously reported, in which no significant statistical relationship was found between infection and sex in cats [27,28]. The lack of association between age and dermatophytosis in this study may be due to the broad age categorization, which may have limited the detection of age-related susceptibility differences, unlike previous studies reporting higher susceptibility in animals younger than 1 year [12].

Environmental exposure may influence fungal carriage and the diversity of cutaneous microbiota and mycobiota in cats. Most cats in the present study were kept exclusively indoors, while only a small number had outdoor access, limiting direct comparisons between indoor and outdoor cats. Outdoor environments may expose animals to a broader range of environmental fungi through contact with soil, vegetation, or other animals. Conversely, indoor environments may impose different ecological pressures, including repeated exposure to household cleaning agents and disinfectants, which may influence microbial communities. Studies conducted in controlled indoor settings, such as hospitals, have shown that repeated exposure of environmental fungi to disinfectants can induce microbial stress responses and may contribute to antifungal resistance [29]. In addition, indoor cats may still acquire environmental microorganisms through close contact with their owners, airborne microbes, or grooming behavior that redistributes microorganisms across body sites. Although these factors were not directly investigated in the present study, they may contribute to differences in fungal ecology between indoor and outdoor environments and warrant further investigation [30].

Our previous study in cats’ external ear canals and facial hairs revealed that *M. pachydermatis* was the most prevalent yeast species (45.80–50.94%) [14,31]. The presence of *M. pachydermatis* in cats showed no significant correlation with filamentous fungi, such as *M. canis* or *Aspergillus* spp., implying minimal direct interaction between yeast and filamentous fungi. However, in humans, the detection of *M. furfur* was significantly associated with a higher prevalence of *Aspergillus* spp., indicating a potential environmental overlap, rather than a direct biological interaction. This association may reflect shared environmental or ecological conditions, including inadequate personal hygiene practices, such as insufficient hand washing, which could facilitate concurrent exposure to multiple fungal species. Individuals with poor hand hygiene may therefore be at an increased risk of fungal colonization or co-detection. However, further investigation is required to understand the clinical significance of this observation.

All fungal isolates tested for antifungal susceptibility in this study were derived from both cats and humans without dermatophytosis lesions; however, the history of prior antifungal drug exposure, whether this was in the form of topical or systemic formulations, could not be tracked or recorded. Because antifungal susceptibility testing for *M. canis* is not yet standardized, the use of various testing procedures may affect MIC values regardless of the original fungal isolate [32]. Our MIC results for *M. canis* were similar to those reported in previous studies. For ITZ, our cat and human isolates exhibited low MIC90 values of 1 and 0.125 µg/mL, respectively, with MICs ≤ 1 µg/mL indicating high susceptibility to ITZ [33]. While ITZ is most recommended for the systemic treatment of *M. canis* in both humans and animals, high MICs have been reported in animals and humans with dermatophytosis, ranging from 4 to 16 µg/mL [34,35]. For TERB, *M. canis* has been reported to be highly sensitive, with MICs ranging from 0.002 to 0.25 µg/mL [36], and the MIC_90_ values of our cat and human isolates were within this range. Terbinafine is highly effective in treating dermatophytosis in cats because it reaches potent, persistent concentrations in the hair that remain well above the MIC90 for weeks after treatment ends [37]. However, various high MICs or strains of *M. canis* that are resistant to TERB have been documented, with MICs ranging from 4 to more than 32 µg/mL in cases of human and animal *M. canis* infections that were refractory to TERB as the initial systemic drug [33,38,39].

The detection of non-WT phenotypes in *A. niger* and *A. terreus* isolates, particularly against ITZ and AMB, is noteworthy among isolates from household and cat-associated sources, with MICs being higher than those generally reported [40,41,42]. Most *A. terreus* isolates exhibit high MICs for AMB and are thus considered intrinsically resistant [43,44]. When *Aspergillus* spp. are resistant or detected as non-WT to ITZ with high MICs, it is typically due to mutations in the cyp51A gene, which encodes the target enzyme for the drug. In particular, strains of *A. fumigatus* exposed to environmental azole compounds undergo structural changes in the enzyme, preventing the drug from binding [45]. It should be noted that *Aspergillus* spp. are primarily environmental molds and are not considered zoonotic pathogens. Their detection on cat hair most likely reflects environmental exposure and transient household mycobiota rather than direct animal-to-human transmission. Nonetheless, when airborne *Aspergillus* spores are present at elevated levels indoors, they can pose a risk of opportunistic infection, especially for susceptible or immunocompromised individuals [45]. While *M. pachydermatis* and *M. furfur* isolates from a similar set of study groups remained largely susceptible to ITZ, KZ, and TERB [14], the transient mycobiota on cat and human bodies associated with the household environment reduced antifungal susceptibility. These findings suggest that pet animals can act not only as reservoirs of zoonotic dermatophytes but also as carriers of strains with reduced antifungal susceptibility, potentially contributing to environmental contamination and exposure within households [46,47,48]. Inappropriate treatment duration, missed or delayed diagnosis, drug pharmacokinetics, the immune status of the animals involved, and the severity and location of the infection may contribute to antifungal resistance [49]. The widespread use of antifungal treatments for dermatophytosis raises concerns about emerging resistance [13,50], indicating the importance of continued surveillance and careful antifungal use in both veterinary and human healthcare.

**Figure 3:**
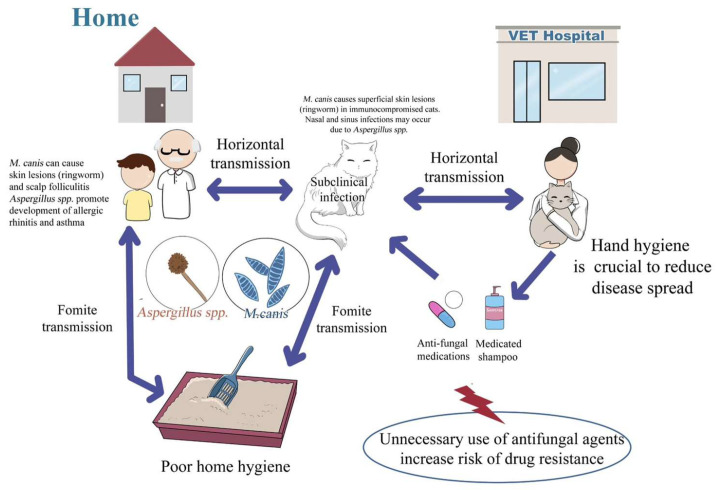
Fungal transmission between cats and humans may occur through direct horizontal or fomite transmission at home and in veterinary environments. Subclinically infected cats serve as reservoirs for *Microsporum canis* and *Aspergillus* spp., which are associated with the development of ringworm and respiratory illness in owners and veterinary staff. Environmental controls, such as proper litter sanitation and hand hygiene, help prevent and manage zoonotic infections. The overuse of antifungals and medicated shampoos can promote drug resistance. Arrows indicate potential transmission pathways.

Taken together, these findings highlight the important role of long-haired cats as potential sources of zoonotic dermatophytes within households and reinforce the importance of preventive hygiene measures, environmental cleaning, and rational antifungal use. These measures are particularly important for protecting vulnerable populations, including children, the elderly, and immunocompromised individuals, within a One Health framework (Figure 3). Fungal infections caused by dermatophytes, such as ringworm and scalp fungi, are common examples of zoonotic diseases. *M. canis* has been found under cat owners’ nails, showing that the fungus can spread to other body parts and cause infection due to scratching. Damp indoor conditions and the presence of cats can elevate levels of airborne fungi such as *Aspergillus* spp., increasing the risk of fungal infections and asthma symptoms in susceptible people [51]. To reduce transmission from cats to humans, proper hand hygiene, regular household cleaning, appropriate cat bathing, use of personal protective equipment in veterinary settings, and rational antifungal use are essential.

## 5. Limitations

This study was conducted in a restricted geographic area, which may limit the generalizability of the findings to regions with differing environmental and cultural contexts. Thailand’s tropical climate may enhance fungal proliferation and influence prevalence patterns as compared with temperate climates. Additionally, variability in cat care practices, including grooming routines, housing conditions, and hygiene management, may affect fungal carriage. Finally, the widespread availability and ease of access to antifungal medications among the public may impact antifungal susceptibility profiles, potentially contributing to the development of reduced susceptibility and complicating the interpretation of resistance-related findings. In addition, the relatively small number of cats with outdoor access in this study (*n* = 7) may have limited the statistical power to detect potential differences in fungal carriage between indoor and outdoor cats. Nonetheless, the present findings have implications for fungal transmission and control.

## 6. Conclusions

The findings show that healthy cats and their owners often share fungal species, suggesting potential household exposure and zoonotic transmission of *M. canis*. Long hair was a significant risk factor for dermatophyte carriage in cats. Antifungal susceptibility profiles were similar between feline and human isolates, but non-WT *Aspergillus* strains indicated emerging resistance. These findings provide baseline surveillance data that may support future One Health monitoring of shared fungal flora and antifungal susceptibility trends at the human–animal interface.

## Figures and Tables

**Table 1:** Prevalence (95% confidence interval) of identified filamentous fungal species from cat facial hairs versus human nails.

Identified Mold Genus	Number of Cats (*n* = 59)		Number of Human Nails (*n* = 59)	
	Positive Culture	Prevalence (%; 95% CI)	Positive Culture	Prevalence (%; 95% CI)
*Aspergillus* spp.	39	66.10 (52.61–77.92)	27	45.76 (32.72–59.24)
*Hyaline septate fungi*	32	54.24 (40.75–67.28)	21	35.59 (23.55–49.13)
*Dematiaceous fungi*	26	44.07 (31.15–57.60)	5	8.47 (2.81–18.68)
*Microsporum canis*	17	28.81 (17.76–42.08)	4	6.78 (1.88–16.46)
*Penicillium* spp.	10	16.95 (8.44–28.97)	2	3.39 (0.41–11.71)

**Table 2:** Association of isolated filamentous fungi species collected from cats’ facial hair and humans’ nails.

Identified Mold Genus	Isolates from Cats	Isolates from Humans		*p*-Value
		Positive	Negative	
*Microsporum canis*	positive	4	13	0.010
	negative	0	42	
*Aspergillus flavus*	positive	6	17	1.000
	negative	9	27	
*Aspergillus niger*	positive	11	15	0.050
	negative	6	27	
*Aspergillus fumigatus*	positive	0	1	0.138
	negative	1	57	
*Aspergillus terreus*	positive	0	1	0.094
	negative	0	58	

**Table 3:** Association test between various lifestyle factors for cats and positive culture results for *Microsporum canis*.

Lifestyle Factors	Number of Cats (%)		*p*-Value
	Positive Culture	Negative Culture	
Hair length			
Short-haired (≤3 cm)	4 (23.53%)	24 (57.14%)	0.024
Long-haired (>3 cm)	13 (76.47%)	18 (42.86%)	
Bathing frequency			
≤1 month	7 (41.18%)	8 (19.05%)	0.103
>1 month or never	10 (58.82%)	34 (80.95%)	
Sex			
Male	10 (58.82%)	27 (64.29%)	0.770
Female	7 (41.18%)	15 (35.71%)	
Age group (*n*)			
≤6 years	10 (58.82%)	25 (59.52%)	1.000
>6 years	7 (41.18%)	17 (40.48%)	
Cat rearing (%; *n*)			
Indoor only	13 (76.47%)	39 (92.86%)	0.176
Outdoor access	4 (23.53%)	3 (7.14%)	

**Table 4:** Prevalence (95% confidence interval) of identified filamentous fungal species from cat facial hairs or external ear canal positive for *Malassezia pachydermatis* and from cat facial hairs or external ear canal negative for *M. pachydermatis*.

Identified Mold Genus	Number of Positive Cats *M. pachydermatis* (*n* = 33)		Number of Negative cats *M. pachydermatis* (*n* = 26)		*p*-Value
	Positive Culture	Prevalence (%; 95% CI)	Positive Culture	Prevalence (%; 95% CI)	
*Microsporum canis*	9	27.27 (13.30–45.52)	8	30.77 (14.33–51.79)	0.7684
*Aspergillus* spp.	24	72.73 (54.48–86.70)	15	57.69 (36.92–76.65)	0.2258
*Penicillium* spp.	6	18.18 (6.98–35.46)	4	15.38 (4.36–34.87)	0.7762
*Hyaline septate fungi*	18	54.54 (36.35–71.89)	14	53.85 (33.37–73.41)	0.9573
*Dematiaceous fungi*	17	51.52 (33.54–69.20)	9	34.62 (17.21–55.67)	0.1943
*Non-septate fungi*	3	9.09 (1.92–24.33)	2	7.69 (0.94–25.13)	0.8481

**Table 5:** Prevalence (95% confidence interval) of identified filamentous fungal species from human nails positive for *Malassezia furfur* and negative for *M. furfur*.

Identified Mold Genus	Number of Humans Positive for *M. furfur* (*n* = 18)		Number of Humans Negative for *M. furfur* (*n* = 41)		*p*-Value
	Positive Culture	Prevalence (%; 95% CI)	Positive Culture	Prevalence (%; 95% CI)	
*Microsporum canis.*	0	0	4	9.76 (2.72–23.13)	0.1699
*Aspergillus* spp.	12	66.67 (40.99–86.66)	15	36.58 (22.12–53.06)	0.0327
*Penicillium* spp.	1	5.56 (0.14–27.29)	1	2.43 (0.06–12.86)	0.5425
*Hyaline septate fungi*	6	33.33 (13.34–59.01)	15	36.58 (22.12–53.06)	0.8102
*Dematiaceous fungi*	2	11.11 (1.38–34.71)	3	7.32 (1.54–19.92)	0.6299

**Table 6:** Antifungal susceptibility of *Microsporum canis* and *Aspergillus* spp. isolates in cats and humans.

Antifungal Drugs	Test Fungi	Isolated from (*n*)	Minimum Inhibitory Concentration (µg/mL)				Susceptibility Phenotypes	
			Range	MIC_50_	MIC_90_	GM ± SD	Wild Type	Non-Wild Type
Amphotericin B	*Microsporum canis*	Cat (17)	0.5–4	1	4	1.4 ± 1.17	ND	ND
		Human (4)	0.5–2	1	2	1.0 ± 0.63	ND	ND
	*Aspergillus flavus*	Cat (10)	1–4	2	4	2.6 ± 1.18	10	0
		Human (7)	1–2	2	2	1.8 ± 0.38	7	0
	*Aspergillus niger*	Cat (14)	0.125–1	0.5	1	0.6 ± 0.30	14	0
		Human (11)	0.125–1	0.5	1	0.5 ± 0.31	11	0
	*Aspergillus* *fumigatus*	Cat (1)	1	ND	ND	ND	1	0
		Human (1)	1	ND	ND	ND	1	0
	*Aspergillus terreus*	Cat (1)	16	ND	ND	ND	0	1
		Human (0)	-	-	-	-	-	-
Itraconazole	*Microsporum canis*	Cat (17)	0.0625–1	0.25	1	0.3 ± 0.33	ND	ND
		Human (4)	0.0625–0.125	0.125	0.125	0.1 ± 0.03	ND	ND
	*Aspergillus flavus*	Cat (10)	0.25–1	0.5	1	0.5 ± 0.24	10	0
		Human (7)	0.25–1	0.5	1	0.5 ± 0.22	7	0
	*Aspergillus niger*	Cat (14)	0.25–8	2	8	2.8 ± 2.97	10	4
		Human (11)	2–8	4	8	3.5 ± 2.70	9	2
	*Aspergillus* *fumigatus*	Cat (1)	1	ND	ND	ND	1	0
		Human (1)	2	ND	ND	ND	0	1
	*Aspergillus terreus*	Cat (1)	1	ND	ND	ND	1	0
		Human (0)	-	-	-	-	-	-
Terbinafine	*Microsporum canis*	Cat (17)	0.25–1	0.25	1	0.3 ± 0.49	ND	ND
		Human (4)	0.125–0.25	0.25	0.25	0.2 ± 0.06	ND	ND
	*Aspergillus flavus*	Cat (10)	0.5–1	1	1	0.9 ± 0.24	ND	ND
		Human (7)	0.25–1	0.5	1	0.5 ± 0.25	ND	ND
	*Aspergillus niger*	Cat (14)	0.5–4	2	4	1.5 ± 2.34	ND	ND
		Human (11)	0.5–8	4	8	2.9 ± 2.84	ND	ND
	*Aspergillus* *fumigatus*	Cat (1)	>8	ND	ND	ND	ND	ND
		Human (1)	>8	ND	ND	ND	ND	ND
	*Aspergillus terreus*	Cat (1)	8	ND	ND	ND	ND	ND
		Human (0)	-	-	-	-	-	-
Ketoconazole	*Microsporum canis*	Cat (17)	0.016–1	0.125	0.5	0.2 ± 0.26	ND	ND
		Human (4)	0.016–0.25	0.063	0.25	0.1 ± 0.10	ND	ND
	*Aspergillus flavus*	Cat (10)	0.25–2	0.5	1	0.9 ± 0.54	ND	ND
		Human (7)	0.5–2	1	2	0.9 ± 0.5	ND	ND
	*Aspergillus niger*	Cat (14)	0.25–4	4	4	2.2 ± 3.26	ND	ND
		Human (11)	2–16	8	8	6.2 ± 1.93	ND	ND
	*Aspergillus fumigatus*	Cat (1)	4	ND	ND	ND	ND	ND
		Human (1)	4	ND	ND	ND	ND	ND
	*Aspergillus terreus*	Cat (1)	4	ND	ND	ND	ND	ND
		Human (0)	-	-	-	-	-	-

MIC_50_ and MIC_90_ values were recognized as the minimum concentrations of each antifungal drug at which 50% and 90% of the growth of fungal isolates was inhibited. GM ± SD: Geometric Mean ± Standard Deviation; ND: not determined.

## Data Availability

The datasets generated or analyzed during this study are available from the corresponding author upon reasonable request.

## Supplementary Materials

The following supporting information can be downloaded at: https://www.mdpi.com/article/10.3390/jof12040245/s1. Table S1: Identified fungal genus and accession numbers.
