# Supplementary material for: Mycological Survey of Fungal Carriage in Cats and Their Owners: Analysis of Species Diversity and Antifungal Susceptibility
Source: J Fungi (Basel). 2026 Mar 26;12(4):245. doi: 10.3390/jof12040245 (PMC13118097; doi:10.3390/jof12040245)
Supplement: Supplementary file 1 [file jof-12-00245-s001.zip › jof-4190082-supplementary.pdf]

Table S1. Identified fungal genus and accession numbers.

Species	Strain	Source	Origin	Accession number
				ITS
M. canis	KU20020.114	Cat	Thailand	PX930861
M. canis	KU20020.126	Cat	Thailand	PX930876
M. canis	KU20020.132	Cat	Thailand	PX930862
M. canis	KU20020.147	Cat	Thailand	PX930877
M. canis	KU20020.178	Cat	Thailand	PX930863
M. canis	KU20020.228	Cat	Thailand	PX930864
M. canis	KU20020.262	Cat	Thailand	PX930865
M. canis	KU20020.314	Cat	Thailand	PX930866
M. canis	KU20020.320	Cat	Thailand	PX930868
M. canis	KU20020.346	Cat	Thailand	PX930905
M. canis	KU20020.408	Cat	Thailand	PX930875
M. canis	KU20020.411	Cat	Thailand	PX930869
M. canis	KU20020.423	Cat	Thailand	PX930906
M. canis	KU20020.429	Cat	Thailand	PX930907
M. canis	KU20020.435	Cat	Thailand	PX930908
M. canis	KU20021.28	Cat	Thailand	PX930871
M. canis	KU20021.32	Cat	Thailand	PX930872
M. canis	KU20020.316	Human	Thailand	PX930867
M. canis	KU20020.350	Human	Thailand	PX930870
M. canis	KU20021.30	Human	Thailand	PX930874
M. canis	KU20021.36	Human	Thailand	PX930873

A. niger	KU20020.294	Cat	Thailand	PX930881
A. niger	KU20020.202	Cat	Thailand	PX930909
A. niger	KU20020.297	Human	Thailand	PX930882
A. niger	KU20020.212	Human	Thailand	PX930910
A. fumigatus	KU20020.250	Cat	Thailand	PX930879
A. fumigatus	KU20020.256	Human	Thailand	PX930880
A. flavus	KU20020.439	Cat	Thailand	PX930886
A. flavus	KU20021.17	Cat	Thailand	PX930884
A. flavus	KU20020.440	Human	Thailand	PX930887
A. flavus	KU20021.19	Human	Thailand	PX930883
A. terreus	KU20020.207	Cat	Thailand	PX930885
